# Green potential of *Pleurotus* spp. in biotechnology

**DOI:** 10.7717/peerj.6664

**Published:** 2019-03-29

**Authors:** Alona S. Sekan, Olena S. Myronycheva, Olov Karlsson, Andrii P. Gryganskyi, Yaroslav Blume

**Affiliations:** 1 Institute of Food Biotechnology and Genomics, National Academy of Science of Ukraine, Kyiv, Ukraine; 2 Division of Wood Science and Engineering, Department of Engineering Sciences and Mathematics, Lulea University of Technology, Skelleftea, Sweden; 3 LF Lambert Spawn Co., Coatesville, PA, USA

**Keywords:** Waste bioremediation, Mushroom cultivation, *Pleurotus*, Degradation, Polycyclic aromatic hydrocarbons, Enzyme, Lignin, Gene, Identification

## Abstract

**Background:**

The genus *Pleurotus* is most exploitable xylotrophic fungi, with valuable biotechnological, medical, and nutritional properties. The relevant features of the representatives of this genus to provide attractive low-cost industrial tools have been reported in numerous studies to resolve the pressure of ecological issues. Additionally, a number of *Pleurotus* species are highly adaptive, do not require any special conditions for growth, and possess specific resistance to contaminating diseases and pests. The unique properties of *Pleurotus* species widely used in many environmental technologies, such as organic solid waste recycling, chemical pollutant degradation, and bioethanol production.

**Methodology:**

The literature study encompasses peer-reviewed journals identified by systematic searches of electronic databases such as Google Scholar, NCBI, Springer, ResearchGate, ScienceDirect, and ISI Web of Knowledge. The search scheme was divided into several steps, as described below.

**Results:**

In this review, we describe studies examining the biotechnological feasibility of *Pleurotus* spp. to elucidate the importance of this genus for use in green technology. Here, we review areas of application of the genus *Pleurotus* as a prospective biotechnological tool.

**Conclusion:**

The incomplete description of some fungal biochemical pathways emphasises the future research goals for this fungal culture.

## Introduction

The large-scale commercial production of edible mushrooms derived from the successful implementation of microbial technology owing to their nutritional, economic, and ecological value and medicinal properties. Notably, the genus *Pleurotus* (Jacq.: Fr.) Kumm. (Pleurotaceae, higher Basidiomycetes) is the second most distributed edible mushroom worldwide and has unique high nutritional value and therapeutic properties. The culture of growing oyster mushrooms was introduced to the West from China and during World War I. Germany successfully developed cultivation methods for the production of *Pleurotus ostreatus* as a new and valuable food source to defeat hunger (Piska, Ziaja & Muszynska, 2016). At present, several species of the genus *Pleurotus* have high commercial value in the global market of edible cultivated mushrooms (Pawlik et al., 2012). The presence of essential amino acids, such as arginine, glutamine, and glutamic acid, as well as vitamins and minerals, is characteristic of *Pleurotus* species (Da Silva et al., 2012). The most popular among them, *Pleurotus ostreatus*, also known as hiratake, is a traditional edible, highly nutritious mushroom with a nutrient-rich dietary composition (Gregori, Svagelj & Pohleven, 2007). This mushroom has high nutritional value due to its high protein, fiber, and carbohydrate contents. Beside obvious importance in the worldwide agriculture and food industry, oyster mushrooms also were considered as an important object for the medical purpose (Moussa, 2009). Secondary metabolites isolated from *Pleurotus* fruiting body and mycelia show strong, versatile health-promoting and therapeutic effects. Valuable bioactive compounds from oyster mushrooms can be divided into two groups with high and low molecular weight (Morris et al., 2017; Golak-Siwulska et al., 2018). A bundle of reports concerning oyster mushrooms brings to light the new substances with such properties as an antibiotic, antitumor, antiviral, and anticholesterolic activities (Selegean, Putz & Rugea, 2009). The radioprotective effects of an extract from *Pleurotus ostreatus* mycelium have been studied by Lauradó et al. (2015) and administered in a prophylactic schedule to Balb/c mice. In general, compounds with pharmacological activities that identified in fungi, stimulate different cell populations of the immune system, such as macrophages, natural killer cells, *T*-cells, and modulate cytokine system as well (Oloke & Adebayo, 2015).

Additionally, oyster mushrooms are low-calorie and exhibit low fat and sodium contents (Patil et al., 2010). Because of low lipid concentration and sugars, *Pleurotus* species are classified as low-energy food products (Kalač, 2016). According to the *Pleurotus* species mineral composition analysis, the most evaluated elements are Cd, Hg, As, and Pb (Deepalakshmi & Mirunalini, 2014). Mostly, fruit body nutritional content depends on what kind of substrate and pollutants concentration is represented. Standard content for mushrooms from unpolluted fields reach on such elements as Al, Zn, Mn, Cd, and others, either (Gucia et al., 2012). On another hand, heavy metals for white-rot fungi, in general, are the critical factor for synthesis of cellulolytic and hemicellolytic enzymes during the biodegradation process. Thereby, the increased attention should be paid to the accumulation of heavy metals in *Pleurotus* mushrooms as a food source (Bellettini et al., in press). However, the ability of *Pleurotus* species to absorb heavy metals from the environment can be helpful in recovery strategies, such as mycoremediation (Alves et al., 2017).

*Pleurotus eryngii* also has established economic importance among the species in this genus. The fruiting bodies of *Pleurotus eryngii* are comparable to those of cultivated oyster mushrooms but possess a distinctly pronounced aroma and superior nutritional quality (Stamets, 2011; Mau et al., 1998) studied the flavor compounds of *Pleurotus eryngii* and found them to consist of mostly volatiles and taste components. Mohamed & Farghaly (2014) described the difference in chemical composition between fresh and dried *Pleurotus ostreatus* and their bioactive secondary metabolites. Among the studied substances, the authors found 107 different metabolites and recommended fresh mushroom fruit bodies as a source of aromatic compounds and dried mushrooms as a source of antioxidants. Ahmed et al. (2008) described the cultivation of *Pleurotus floridanus* (Singer, 1948) on different straw wastes, such as soybean, paddies, and wheat straws, and their combinations to determine the effects of the straw substrate additives on yield, moisture content, and crude protein. In perspective, usage of those substrate formulas could be considered as a basis for the cultivation of *Pleurotus florida* regarding raw material abundance for large-scale production. Cultivation of those species is basically an adaptation of their mycelial growth and fruiting to existing raw materials as the development of substrate-processing technologies.

In contrast, the regulation of fruit body nutritional content can be acquired via the application of different agricultural wastes with different chemical compositions and supplements. Additionally, the selective properties of *Pleurotus* enzymatic systems are desirable for applications in biomass degradation and the production of derivatives for green chemicals and biofuel. The principal moments of *Pleurotus* application and emphasise some informational gaps that define “critical” areas of research and their relevance to the industrial sector investigated in this study.

## Survey Methodology

The literature study encompasses peer-reviewed journals identified by systematic searches of electronic databases such as Google Scholar, NCBI, Springer, ResearchGate, ScienceDirect, and ISI Web of Knowledge. The search scheme was divided into several steps, as described below.

First, we focused on technological and nutritional fungal properties, the primary fungal enzymes, and examples of biotechnological applications, while collecting the primary information. Thus, every selected category was used in combination with a comparable keyword reflecting the review questions. Papers with a high citation rate in Google Scholar (180 peer-reviewed articles) were collected in an initial database to gain an overview of the general research interest in that area. The dates were identified as the timeframe for the analysis.

Categorization and research relevance, number of subsequent citations, and journal impact factor were then used to verify and compare the content from research databases such as NCBI, Springer, ResearchGate, ScienceDirect, and ISI Web of Knowledge. An expert analysis was applied to validate the competence of the selected articles and optimize the structure. A total of 121 articles were chosen and organized into the list by Zotero (http://www.zotero.org/) to obtain the file extension .ris for applications using the software for further analysis.

Finally, we conducted and visualized the bibliometric map using the software VOSviewer (http://www.vosviewer.com/) to cluster related and frequently occurring keywords and terms. According to the co-cited terms, the visualization density map for collected articles lists most of the frequently observed keywords, such as *phylogeny, genetic variability, environmental microbiology, mushroom cultures, lignin oxidation, mushrooms’ bioactive proteins, straw pretreatment*, and *mycelium assessment*. In order to do that, the main article was developed to elucidate current knowledge gaps and further identify research applications for the genus *Pleurotus* in biotechnology.

## Results

### Taxonomic identification of *Pleurotus* spp.

The “life story” of *Pleurotus* spp. is an excellent example of the domestication of a common fungal saprotrophs from forest ecosystems (Pathak, Joshi & Dwivedi, 2009), (Vilgalys & Sun, 1994). *Pleurotus* spp. belong to the family *Pleurotaceae*, which contains six genera with a total of 94 species (Hawksworth et al., 1996). The genus *Pleurotus* contains 30 species and subspecific taxa of *Pleurotus ostreatus* (Venturella, Gargano & Compagno, 2015). Some species, such as *Pleurotus cystidiosus* or *Pleurotus dryinus,* have been shown to undergo the asexual sporulation during their life cycle, which is uncommon for *Pleurotaceae*. Therefore, industrial cultivation of such species is considered insufficient (Shnyreva et al., 2017).

*Pleurotus* mushrooms have enormous potential for the biotechnological degradation of lignocellulosic materials and some organic pollutants (Sánchez, 2010). The most notable feature is an ability to selectively biodegrade lignin by exposes celluloses and hemicelluloses (Kerem, Friesem & Hadar, 1992; Elisashvili et al., 2008; Bánfia et al., 2015). In contrast, many species of the genus *Pleurotus* have been reported to participate in numerous interactions with microorganisms, plants, or animals (Tsuneda & Thorn, 2011). For example, *Pleurotus eryngii* can live as a saprobe and a parasite on the roots of umbelliferous plants, for example, family *Apiaceae* (Hilber, 1982). In the past, fungal population growth on host-plant roots or the lower parts of stems, separately or in small groups arising from the same location, has been complicated by the simultaneous identification of several taxa, leading to ambiguity of the obtained data and challenging interpretations. In the last century, the determination of fungal taxonomic status has been based mainly on the characterization of morphological features.

*Pleurotus* fruitbodies are identified based on their unique morphological characteristics, such as the shape, color, and size of the hymenophore. However, this strategy can sometimes lead to inaccurate taxonomic conclusions (Vilgalys et al., 1996), (Choi, Ding & Cha, 2007). Hence, the importance of elucidating the *Pleurotus* taxonomy at the molecular level is evident. Cultivated fungal varieties can lose their genetic diversity through inbreeding events (Urbanelli et al., 2007), which may lead to the loss of valuable biotechnological properties in newly introduced hybrids that lack species-level identification markers. Thus, it is essential to identify *Pleurotus* cultivars during any period of hyphal development by applying DNA-based analyses of molecular markers, such as simple sequence repeats (Ma et al., 2009), random amplified polymorphic DNA (Yan & Jiang, 2005), and amplified fragment length polymorphism (Urbanelli et al., 2007), and sequence analyses of mitochondrial small subunit ribosomal ribonucleic acid (SSU rRNA) (Gonzalez & Labarère, 2000), cytochrome oxidase genes (*cox*) (Seifert et al., 2007; Nguyen & Seifert, 2008), and partial Elongation factor 1-alpha (*ef1α*) and RNA polymerase II (*rpb2*) genes (Estrada, Jimenez-Gasco & Royse, 2010). Additionally, the fungal mitochondrial genome (mtDNA) is widely used not only for classification purposes, but also to reveal mitochondrial origination and horizontal gene transfer events (Seif et al., 2005; Seifert et al., 2007; Wang et al., 2008). Morphologically, fungi share typical characteristics, that is, the ability to produce asexual arthrospores on basidiomata or on vegetative mycelium (Zervakis, Moncalvo & Vilgalys, 2004). The original study of the sexuality of *Pleurotus* fungi was reported by Vandendries (1933). Additionally, a high percentage of polymorphic loci and an increased genetic distance in *Pleurotus eryngii* relative to other *Pleurotus* spp. were demonstrated by Zervakis, Sourdis & Balis (1994). Concurrently, the classification of *Pleurotus* spp. based on phylogenetic analyses of small mitochondrial subunit ribosomal (*rns*) genes has also been described in several reviews (Garber & Yoder, 1983; Gonzalez & Labarère, 2000). Furthermore, the phylogenetic relationships among closely related species of genus *Pleurotus* have been investigated through cladistics analysis using the V4 domain of mitochondrial *rns* (Bao, Aimi & Kitamoto, 2005). Also, the phylogeny of this genus was constructed by rRNA gene cluster analysis based on internal transcribed spacer sequences of rDNA. The phylogenetical tree was designed for 31 *Pleurotus* strains of different origin and 10 reference sequences from GenBank (Shnyreva & Shnyreva, 2015). A great example of using phylogenetic analyses for *Pleurotus ostreatus* species complex for the industrial purpose demonstrated on report of Li et al. (2017). Seven different oyster mushroom lineages were identified from a pool of 284 samples with different commercial names gathered from different mushroom spawn preservation centers, companies, and field isolations. For Li et al. (2017), the *rpb2* gene is the most useful marker for *Pleurotus ostreatus* lineage identification among four promising barcode genes they used.

Clustering analysis was performed for two closely related species (17 strains) of *Pleurotus ostreatus* that show identical restriction polymorphism (RFLP) types. As reported by Wang et al. (2008), the complete mitochondrial genome has a circular shape and a size of 73,242 bp encompassing 44 known genes encoding 18 proteins and 26 RNA genes. According to the complete genome sequence of *Pleurotus ostreatus*, nine genes from the manganese peroxidase (MnP-) and VP-encoding (versatile peroxidase) gene family were identified to play significant roles in the degradation of some biopolymers like lignin (Salame et al., 2014). Investigation of the expression of certain MnP/VP genes identified the complex work for both of them in the ligninolytic system (Nakazawa et al., 2017). Additionally, mutation in *chd1‐1* gene and targeted disruption of *wtr1* gene leads to the ability of *Pleurotus ostreatus* to biodegrade wood lignin. These genes are valuable for biotechnological use, such as the processing of agricultural waste.

### Lignin degradation by *Pleurotus* spp.

Lignin is the second most abundant plant biopolymer in world ecosystems after cellulose. It is an aromatic polymer and one of the significant components of plant secondary cell walls, providing plant cells with rigidity, water impermeability, and resistance to microbial attack. The irregular chemical structure of this polymer imposes special restrictions on its ability to undergo biodegradation (Kirk & Farrell, 1987). In general, the worldwide lignocellulosic biomass of different kinds of crops is produced in large amounts of approximately 73.9 Tg/year and leads to waste utilization problems (Kim & Dale, 2004). In contrast, the waste biomass has a high potential for further production of biotechnological products, such as bioethanol, biogas, and other useful chemicals. One of the main issues is the derivatisation of heterogeneous waste biopolymers, such as lignin, cellulose, and hemicellulose. To date, numerous reports have described different methods of lignocellulosic biomass pretreatment of agricultural wastes using physical, mechanical, and chemical mechanisms. Enzymatic systems from various microorganisms target lignin matrix decomposition and the release of other lignocellulose structure monomers, oligos, and polymers (Guerriero et al., 2016). Applications of white-rot fungi are the most popular because their pure cultures can mineralize lignin fibers from a substrate into CO_2_ and water (Hatakka, 1983; Hatakka & Hammel, 2011; Isroi et al., 2011). However, lignin is not a target source of energy or a substrate of primary metabolism for white-rot fungi. The decomposition of lignin is aimed at the gain of cellulose and hemicellulose, which are energy-rich substances (Bazanella et al., 2013). Fungal biodegradation of lignin mostly is a result of the oxidation process with peroxidase and laccase. Peroxidase isoenzymes are present in almost all living organisms and catalyze many different important for life reactions. This kind of ferments is also applying for industrial purpose. Recently was identified novel VP from the fruiting bodies of *Pleurotus pulmonarius* that differs those of mushroom peroxidases (Zou, Wang & Zhang, 2018). The difference of peroxidases from liquid cultures of *Pleurotus eryngii* was confirmed by comparison for N-terminal sequence and peptide mapping analysis. According to the description, this novel enzyme has a high potential for future application in treatment and utilization of agricultural wastes.

White-rot fungi can quickly and non-specifically degrade lignin polymers in woody tissues (Eriksson, Blanchette & Ander, 1990; Camarero et al., 1999; Wesenberg, Kyriakides & Agathos, 2003). *Pleurotus* species produce MnP and VP enzymes that provide high adaptability for growth and fruiting for a wide variety of agricultural and industrial types of lignocellulosic disposal (Mikiashvili et al., 2006; Fernández-Fueyo et al., 2014). However, successful enzymatic activity and lignin degradation depend on many factors, such as the fungal strain, nutrient composition of the substrate, moisture content, and pH, among others (Snajdr & Baldrian, 2007). A recent investigation of the lignin-degrading capacity for five white-rot fungi, *Phanerochaete chrysosporium, Pleurotus ostreatus, Lentinus edodes, Trametes versicolor*, and S22 was reported (Wu, Xiao & Yu, 2005). According to the authors, mushrooms were individually used to treat black liquor from a pulp and paper mill. Among all five species, over 71% of lignin and 48% of chemical oxygen demand were removed from the wastewater. Another report described the utility of paper mill as a lignocellulose-based substrate for *Pleurotus* cultivation and shown the strong dependence between mushroom’s growth and medium composition (Skočaj et al., 2018). The highest peroxidases specific activities were detected at days 34 and 40 of the incubation period. During the fermentation, extracellular enzymes with different activities were extracted. They were determined to four groups: cellulase, xylanase, lipase, and peroxidase. The authors demonstrated the capacity of solid wastes as a good substrate for the production of commercially interesting enzymes. Moreover, with the strong of worldwide paper waste and the minimization of lignocellulosic volume toward decreasing the ecological pressure, this strategy of *Pleurotus* application is very promising.

Myronycheva et al. (2017) has shown that temperature sensitivity, vegetative growth, and the generative response are factors affecting the successful cultivation of several oyster mushroom strains grown under conditions typical of European climate zones, indoors on agricultural waste. According to that report, some *Pleurotus ostreatus* strains exhibited differences in response to variations in climatic and substrate conditions. Hence, flexibility in both vegetative growth and fruit body formation suggests a high potential of the presented *Pleurotus* strains for commercial purposes. Required fungal features can be enhanced through alterations of external and inner conditions.

### Manganese peroxidase

An important factor affecting biodegradation processes is the presence of metals in the substrate. One report (Baldrian et al., 2005) determined the possible effects of some heavy metal ions on the process of wheat straw degradation by *Pleurotus ostreatus*. Moreover, one of the main toxic elements spread into the environment is cadmium because it inhibits many of natural processes (Bellettini et al., in press). During biodegradation on the wheat straw substrate, *Pleurotus ostreatus* produces enzymes, such as endo-1,4-β-glucanase, exo-1,4-β glucanase, 1,4-β-glucosidase, endo-1,4-β-xylanase, 1,4-β-xylosidase, endo-1,4-β-mannanase, and 1,4-β-mannosidase, as well as the ligninolytic enzymes MnP and laccase. Additionally, the effect of copper and cadmium with further increases in laccase activity in liquid cultures of *Pleurotus ostreatus* has been described (Palmieri et al., 2000). The authors observed a loss of dry mass and reduced rate of extractable phenolic compounds in the presence of Mn, and low hydrogen peroxide concentrations indicated higher consumption of MnP during the catalytic cycle.

Due to the ability of *Pleurotus* species to produce one or several lignin-degrading enzymes, they are efficient in breaking down synthetic dyes. Manganese peroxidase (MnP, EC 1.11.13) can oxidise natural substrates with subsequent depolymerisation of lignin or recalcitrant xenobiotics to produce aromatic radicals (Van Aken et al., 1999; Camarero et al., 2000), as well as phenols and other small redox potential compounds (Hildén et al., 2005). One study (Sarkar, Martínez & Martínez, 1997) described the catalytic properties of MnP isoenzymes extracted from *Pleurotus ostreatus* growing in a liquid medium with peptone. The authors also emphasised the significance of the medium composition because the N-terminal sequence of the *Pleurotus ostreatus* isoenzyme differed from their previously published sequence of MnP extracted from these fungi growing in liquid medium with ammonium tartrate (Martínez et al., 1996). Additionally, MnP extracted from *Pleurotus ostreatus* had the same pl (3.75) and N-terminal sequence as MnP1 isolated from *Pleurotus eryngii*.

In addition to the ability to interact with Mn2+ as an ion donor, some MnPs also oxidise phenolic and non-phenolic aromatic compounds (e.g., veratryl alcohol) under certain conditions (Hofrichter, 2002). This kind of enzymatic property has been described for *Pleurotus eryngii* and a few other white-rot fungi (Böckle et al., 1999). In another review (Giardina et al., 2000), the production of two different MnP isoenzymes—MnP2 and MnP3—by *Pleurotus ostreatus* was examined during the growth cycle on wood sawdust as the solid substrate. Moreover, oyster mushroom growth has been shown to be accelerated on fir sawdust compared with poplar, but with reduced production of MnP. MnP can also be involved in the degradation of bisphenol A (2,2-bis(4-hydroxyphenyl)propane, BPA), an endocrine-disrupting chemical produced by *Pleurotus ostreatus* (Hirano et al., 2000). Over 12 days of mycelial growth, a BPA content of 80% was reduced by MnP. Bisphenol A is metabolised to phenol, 4-isopropenylphenol, 4-isopropylphenol, and hexestrol. Enhanced expression of fungal MnP as an efficient degrader of lignin and different pollutants represents an attractive tool either for bioconversion or waste utilisation (Irie et al., 2001). The recombinant MnP isozyme MnP3 in *Pleurotus ostreatus* was expressed using a DNA-mediated transformation system consisting of a molecular breeding approach. The transformation of oyster mushroom was performed using the PEG/CaCl_2_ method (Honda et al., 2000). An MnP-overproducer was isolated from *Pleurotus ostreatus* transformants containing recombinant *mnp3* constructs under the control of *sdil* expression signals with a carboxin-resistant vector plasmid pTM1. The transformed fungi showed several times higher levels of MnP activity than the wild-type control strain during the early stage of liquid culture.

### Laccase

Another type of ligninolytic enzyme produced by *Pleurotus* spp. is represented by the laccases (benzenediol: oxygen oxidoreductases, Lac, EC 1.10.3.2)—copper-containing oxidases that act on phenolic substrates by catalyzing the oxidation their phenolic hydroxyl groups to phenoxy radicals while dioxygen (O_2_) is reduced to water (Bourbonnais et al., 1995; Wong, 2009; Christopher, Yao & Ji, 2014). Laccases are broadly functional enzymes involved in lignin biosynthesis, the process of pigment formation during fungal sporulation, plant pathogenesis, iron metabolism, and kernel-browning processes in plants (Hoopes & Dean, 2004; Higuchi, 2004). Moreover, laccase, as a glycoprotein enzyme that is expressed by white-rot fungi and participates in the carbon cycle, together with other ligninolytic fungal enzymes, can degrade lignin, which is one of the principal components of wood (Leonowicz et al., 2001; Widsten & Kandelbauer, 2008). Genes for this enzyme have been identified in both ascomycetes and basidiomycetes. Comparison of laccase gene sequences from different fungi has revealed a high degree of identity among them (Thurston, 1994; Soden & Dobson, 2001). The strong oxidative capabilities attract researchers’ attention for these enzymes. The biotechnological potential of those enzymes has thoroughly studied for more than 20 years. A wide range of recent articles described progress in laccase engineering with further application perspectives for numerous issues. Thus, through site-directed mutagenesis, a mutant of *Pleurotus ostreatus* strain was obtained with the most thermostable enzyme among all laccases produced by other known *Pleurotus ostreatus* strains (Autore et al., 2009). The substrate composition, as a source of carbon and organic nitrogen, plays a vital role in laccase production. Thus, during the cultivation of *Pleurotus sajor-caju* strain PS-2001 in liquid culture with fructose or glucose as carbon sources, the enzymatic activity was found to be 37 and 36 U·mL−1, respectively (Bettin et al., 2009). Furthermore, because of the absence of results with respect to the production of phenoloxidases by *Pleurotus* in medium containing a low-cost nitrogen source, authors studied the application of casein. Pure casein as a source of organic nitrogen was the most appropriate compound hence, during the cultivation of fungi in medium with sucrose and casein, laccase at 13 U mL−1 was obtained.

Additionally, the presence of silicon-based antifoam and/or Tween 80 at low concentrations had no significant influence on enzyme formation.

On another report, the increasing of *Pleurotus ostreatus* CP-50 fungal culture growth was related to the influence of the carbon and nitrogen sources in the medium (Tinoco et al., 2011). Implementation of additional carbon and nitrogen amount in malt growth medium showed the decreasing of specific laccase production (U/mg biomass). However, fungal culture growth and laccase volumetric activity were increased in four and six times. It could be explained that the sugars are efficient and fast utilized by fungi substrate that induce the laccase activity for biomass production. Extracellular laccase production and laccase isozyme regulation in *Pleurotus sajor-caju* studied via modification of physiological conditions, (Soden & Dobson, 2001). The authors identified four unique laccase isozyme genes that showed a high degree of similarity to laccases from other basidiomycetes and average identities with ascomycete laccases (24–62%). The results indicated that *Pleurotus sajor*-*caju* laccase isozyme genes differentially regulated at the transcriptional level in response to metals such as copper, manganese, and nutrient nitrogen, among others. As an example, the great laccase producing was found on copper sulphate induced solid-state fermentation medium for *Pleurotus ostreatus* PH-1 (Patel et al., 2014). The presence of copper was detected as a central ion in the presence of iron, zinc, and copper laccase purification. Also, it has been shown the increasing of laccase and isoenzymes synthesis in the presence of copper (Baldrian & Gabriel, 2002) in another report, the same *Pleurotus sajor-caju* strain PS2001 was examined for dye-decolorizing ability and polyphenol degradation in liquid culture (Munari, Aparecida Gaio & Dillon, 2009). Pulp and paper mill, as residues of paper manufacturing, were used as substrates with high lignin levels, endowing the effluents with a specific brownish color. The fungal capacity for residue degradation of 90% of the raw effluents from the medium oxygen delignification and bleaching stages were used in the study. The addition to the substrate up to 10% of the mineral solution and different levels of glucose as substrate gives higher yield. The liquid culture of *Pleurotus sajor-caju* effectively reduced the levels of total polyphenols and amounts of dye in residues from the paper industry. Effluent degradation was obtained in the presence of ligninolytic enzymes such as laccases and peroxidases, but the authors did not detect enzymatic content production by the fungus (Munari, Aparecida Gaio & Dillon, 2009). However, the study of Stajić et al. (2006) clearly demonstrated that laccase and peroxidases production depends on the species and strains of the genus *Pleurotus*, conditions of cultivation, and carbon sources as well as nitrogen sources and concentrations.

In general, laccases catalyse the oxidation of a range of aromatic compounds, such as acrylamines, aminophenols, or diphenols. Report by Munari et al. (2008) demonstrated the ability of *Pleurotus sajor-caju* PS2001 to undergo dye decolourisation, which shown for nine anthraquinone-type industrial textile dyes using the agar plate method to achieve a final dye concentration of 100 mg L^−1^. Additionally, the mushrooms were cultivated under conditions of solid-state fermentation as well as liquid culture separately. The solid-state cultivation substrate contained pine sawdust and wheat bran to obtain the enzymatic extract. Enzymatic extracts from solid-state cultures were used to determine laccase and manganese-peroxidase activities and further test their capacity to degrade the textile dyes. Hence, the *Pleurotus sajor-caju* PS2001 strain was shown to have the potential for use in the degradation of textile dyes in residue manufacturing.

The ability of laccase to degrade some polyaromatic hydrocarbons (PAHs) produced by blue laccase from *Pleurotus ostreatus* D1 (BLPO) in the presence of conventional synthetic mediators has been described by Pozdnyakova et al. (2006). To study PAH-degrading compounds containing three to five aromatic rings in the presence of mediators such as ABTS (2,2-azino-bis-(3-ethylbenzthiazoline-6-sulphonic acid) diammonium salt), syringaldazine (4-hydroxy-3,5-dimethoxybenzaldehyde azine), 2,6-dimethoxyphenol, and catechol were used. According to the results, ABTS was a better mediator of anthracene oxidation by BLPO and HBT (1-hydroxybenzotriazole) and a better mediator of fluorene oxidation. Concurrently, pyrene and anthracene were degraded more rapidly in a mixture than separately. Thus, the degradation ability of BLPO depends on the structure of the PAH molecule, type of organic solvent, presence and type of detergent, enzyme concentration, and duration of the reaction.

### Versatile peroxidase

Another type of ligninolytic peroxidase called versatile peroxidase (VP, EC 1.11.1.16) is found in some *Pleurotus* and *Bjerkandera* species (Camarero et al., 1996, 1999; Martínez et al., 1996; Mester & Field, 1998; Moreira et al., 2005) and exhibits activity on aromatic substrates. VP oxidises Mn2+, as MnPs, phenolic and non-phenolic substrates that are typical for LiPs, related peroxidases (e.g., horseradish peroxidase), and generic peroxidases (low redox-potential peroxidases of plant and fungal origin). The structure of VP enzymes is closer to LiP than to MnP isozymes. Moreover, VP can directly oxidise high-redox potential compounds like some industrial dyes, while LiP shows catalytic activity only in the presence of redox mediators. According to the study by Morales et al. (2012), two catalytic sites are responsible for low-redox potential dyes and phenol oxidation. High-redox potential substrate oxidation by VP occurs because VP site Trp-164, as well as a low-efficiency site involved in the oxidation of the same phenols, is localized at the entrance of the heme-distal pocket of the VP molecule. However, a natural oxidizing substrate, H_2_O_2_, leads to reduced stability of the VP molecule and prevents the broad use of the enzyme in industrial and environmental applications (Martínez et al., 2009). Thus, Sáez-Jiménez et al. (2015) studied different ways to improve VP stability in the presence of H_2_O_2_. Primary results were gained by substitution using site-directed mutagenesis of amino acid residues located at specific positions near the heme group affecting the formation, stabilization, and decomposition of Compound III. Hence, the development of a modified biomolecule made this enzyme an attractive industrial tool for further improvement of the molecular structure in response to changes in temperature and an alkaline pH (Garcia-Ruiz et al., 2012).

Tetraterpenoid compounds, which are derived differently from carotenoids, are synthesized for biotechnological application and have attracted increasing interest in the fragrance industry. A new approach to the introduction of a secreted wild-type VP in submerged cultures of *P. sapidus* for β-carotene degradation proposed by Schüttmann et al. (2014). As nitrogen and carbon sources, residues from biogas plants used. The enzyme was purified and characterized biochemically with further cloning of the encoded cDNA due to the heterologous expression of VP in yeast *Hansenula polymorpha*. According to bioinformatics analyses of VP sequences, an open reading frame of 1,083 bp found with 90% similarity to VPs from *Pleurotus eryngii*. A heterologous enzyme successfully produced with an activity of 450 ± 20 mU mg−1 by culturing *H. polymorpha*.

Solid-state cultures of *P. eringii* on the banana peel/skin and other lignocellulose residues such as growth medium were used to evaluate the feasibility of agricultural residue remediation by VP (Palma et al., 2016). Glucose-based liquid medium for comparison of the catalytic parameters of the produced enzyme has used as a conventional source of VP production. The enzymatic activity of VP generated by solid-state culture was detected after 18 days of cultivation and determined to be 10,800 U L−1 (36 U g−1 of the substrate), whereas the enzymatic activity of VP from liquid culture was only 1,800 U L−1. The obtained results included the H_2_O_2_ inhibitory effect and were observed for the enzymes produced in both media. However, the reaction rates for VP synthesized by solid-state cultures showed a reduced impact. VPs were also successfully applied for the degradation of different dyes, both with low-redox and high-redox potential, as a powerful tool in bio-decolourisation. Thus, the ability to decolourise different azo dyes has been shown for VPs isolated from *Pleurotus eryngii* and *Bjerkandera adusta* (Camarero et al., 1999). One report (Pozdnyakova et al., 2015) compared the catalytic properties of VPs produced by *Pleurotus ostreatus* strain D1 and *Bjerkandera fumosa* strain 137. The decolourisation activities of both enzymes were tested on a wide range of dyes containing condensed aromatic rings, such as anthraquinone and anthracene dyes. Both peroxidases rapidly decolourized the anthraquinone dyes with subsequent formation of polymerisation reaction products. In contrast, anthracene-type dyes were degraded very slowly by both tested enzymes. Hence, the differences between dye structures significantly affected the manner of decolourization, probably due to the different electron distribution, charge densities, or steric factors. In the case of overexpression of the VP, MnP2 may lead to improved enzymatic properties. Thus, enhanced productivity of VP MnP2 was gained by using DNA-transformation technology consisting of a molecular breeding approach for isolated *Pleurotus ostreatus* strains (Tsukihara et al., 2006). The recombinant plasmid was introduced into wild-type oyster mushroom using the PEG/CaCl_2_ method, and recombinant strains overexpressing VP MnP successfully obtained. Screening of the obtained recombinants was conducted in the presence of Poly R-478 due to the decolourisation of dye on agar plates in the absence of Mn2+. Additionally, benzo(a)pyrene-removing activity by treatment with recombinant fungal strains also analyzed. According to this report and numerous other relevant studies, VP enzymes are highly attractive industrial tools due to their ability to oxidise different substrates under altered environmental conditions (Knop et al., 2016). However, along with the presence of multiple active sites exhibiting a wide range of VP activities, the potential of the enzymes depends on a variety of factors.

### Waste biodegradation

In the past, mushrooms had not received much attention as a powerful biotechnological tool due to the relatively long duration of biomass pretreatment as well as substantial losses of cellulose and hemicellulose contents during processing (Balan et al., 2008). Despite these deficiencies and after meticulous assessments of fungal enzymatic systems, which have described in many reports, the total duration of biotechnological processes has become much shorter, and sugar loss can be minimized through the use of white rot fungi. Here, *Pleurotus* spp. produced a range of enzymes that are required in biotechnological processes and provide successful applications of fungi for the biodegradation of cellulose-containing substrates: cotton stalks, sugarcane fiber, wheat, or rice straw, among others. This tremendous biotechnological potential was found for the cultivation of the genus *Pleurotus* without requiring the presence of a composting or casing layer and without grinding the substrate and mixing it with water. The substrates used in each region usually depend on locally available agricultural wastes. Since *Pleurotus* spp. can efficiently decompose lignocellulose without chemical or minimal biological pretreatment, a large variety of lignocellulosic wastes can be utilized and recycled (Cohen, Persky & Hadar, 2002).

The preparation of *Pleurotus* substrate from shredded wheat straw has become a routine practice. Wheat straw is the most abundant agricultural residual in the territory of Europe and the second after rice straw on a global scale (Kim & Dale, 2004). In earlier reports (Müller & Trösch, 1986), a range of different basidiomycetes, mostly white-rot fungi, have been studied and tested to identify species that are capable of efficient biogas (methane) production. Hence, Müller and Trösh studied 22 different fungal species grown on wheat straw. Among them, *Pleurotus floridanus* showed more rapid delignification of straw waste. The methane yield was twofold higher than the total amount of straw without pretreatment and huge effect on digestibility of pretreated by fungi biomass has a microbial consortia in the process (Zhong et al., 2011). In the next study of biogas production (Vasmara et al., 2015; Marchetti, Vasmara & Florio, 2016), lignolytic white-rot fungi and cellulolytic or xylanolytic fungi were compared regarding biogas production. Pretreated wheat straw with or without pig slurry during the co-digestion process was used as a substrate. The authors reported a maximum gas production with shorter reaction time for co-digestion compared with fermentation during monoculture digestion. It was shown that the efficiency of pig slurry utilization as a hydration medium for anaerobic digestion can be used for higher bioethanol production.

The efficiency of wheat straw polymer decomposition by *Pleurotus* spp. has been reported with use of commercial cellulose as a standard to compare the release of glucose monomers from untreated straw with that from straw pretreated with *Pleurotus* solid-state fermentation. In that study, lignin content decreased by 51% during the incubation period (90 days) (Kempken, 2013). Also, sugarcane is an excellent substrate for the cultivation of *Pleurotus* species. To date, great attention has focused on this plant as an efficient source of raw material for bioethanol production. The lignocellulosic matrix of sugarcane contains mainly cellulose and hemicellulose as polysaccharides that are suitable for hydrolysis for further production of ethanol and other chemicals. Camassola & Dillon (2009) reported the use of *Pleurotus sajor-caju* PS 2001 in the pretreatment of sugarcane bagasse. The obtained biomass subsequently utilized in the production of cellulases and xylanases by *Penicillium echinulatum.*

Cotton is another biotechnological crop that is distributed worldwide and generates a significant amount of local agricultural waste. Cotton stalks as a substrate have successfully utilized for commercial *Pleurotus* cultivation (Silanikove, Danai & Levanon, 1988). Thus, in the studies of *Pleurotus* growth (Hadar et al., 1992) used cotton stalks because of their fibrous structure, which is similar to hardwood and creates challenges for agrotechnical utilization. During 4 weeks of solid-state fermentation, they detected a significant decrease in lignin content and increased in digestibility in vitro. Ruminants consumed the fermented product at a level of up to 40% of their diet. In another report (Huttermann et al., 2000) described the process for the recycling of agricultural wastes by *Pleurotus* spp., which included the pasteurization of wet straw by solar heat, treatment of wet straw with detergents, and amendment of straw with wastes from the food industry, such as potato pulp and tomato pomace. The methods were suitable for farming applications with low energy consumption. In vitro and in vivo studies reported that the biological treatment with *Pleurotus* species improves the digestibility of roughage for animals because of enzymatic modification effect on cellulose and lignin. In feeding experiments, rams fed with fungus-treated straw exhibited an increase in body weight. The nutritive value of agricultural residues, such as rice straw or corn stalk, was improved by the fungal treatment.

In comparison to chemical approaches like acid hydrolysis, the application of fungal hydrolytic systems is one of the most fruitful and conventional methods in waste utilization technology (Galbe & Zacchi, 2007). Combined technology, in general, includes four steps: pretreatment waste mass for the preparation of cellulose for further enzymatic hydrolysis; enzyme production; enzymatic saccharification of the pretreated waste mass to fermentable sugars; fungal or chemical conversion of the obtained sugars to the final product (e.g., biofuel) (Tian, Fang & Guo, 2012). Rice straw is another agricultural crop that is a vital source of lignocellulose biomass. One report (Balan et al., 2008) described the treatment of rice straw with *Pleurotus ostreatus,* followed by ammonia fibre expansion (AFEX). This pretreatment led to significantly elevated levels of glucan and xylan conversion under less severe AFEX conditions compared with the treatment of rice straw waste directly with AFEX. The main component of rice straw is cellulose, which can be hydrolyzed into glucose with further conversion into bioethanol or biogas.

Mustafa, Poulsen & Sheng (2016) recently described a new approach to methane production by comparing two groups of fungi, ascomycetes (*Trichoderma reesei*) and basidiomycetes (*Pleurotus ostreatus*), to improve the biological degradability of rice straw waste and increase methane production via solid-state anaerobic digestion. During the pretreatment period, *Pleurotus ostreatus* caused significant degradation of the lignin component of straw waste. Simultaneously, this fungus had a limited effect on cellulose degradation. However, by comparison, the application of *Trichoderma reesei* provided better results for lignin and hemicellulose removal. Hence, lignin degradation by *Pleurotus ostreatus* was 33.4% with selectivity (lignin/cellulose removal ratio), resulting in a 120% increase in methane yield compared with untreated rice straw. After applying *Trichoderma reesei*, 23.6% lignin removal and a 78.3% increase in methane yield achieved.

Utilization of food residues is a dominant issue for global foodstuff consumption and has great potential for biotechnological retreatment processes. Banana mostly consumed fruits in India and one of the largest commodities worldwide. This crop generates the potential mass of available agricultural waste (Centre for Monitoring Indian Economy (CMIE), 2001), and banana residuals mainly consist of lignocellulose material suitable for the growth of white-rot fungi. Two species, *Pleurotus ostreatus* and *Pleurotus sajor-caju*, were used for the bioconversion of the banana leaf and pseudostem biomass to investigate their ability to produce ligninolytic and cellulolytic enzymes for solid-substrate fermentation (Reddy et al., 2003). Both *Pleurotus* species showed similar levels of enzymatic activities and patterns of production. However, deficient concentrations of cellulolytic enzymatic activities observed during the process. The authors also demonstrated the dynamics of extracellular protein formation produced by both fungi during biomass degradation over a 40-day period. Another *Pleurotus* species, the white-rot fungus *Pleurotus dryinus* IBB 903 isolated and identified in Georgia, has also displayed high activities of all studied lignocellulolytic enzymes. This strain can also produce a range of enzymes for the submerged fermentation of mandarin peels and tree leaves (Elisashvili et al., 2006).

### Organopolutant biodegradation

Bioremediation is the process of the biological conversion of hazardous wastes to harmless compounds, or to levels that are below dangerous level. Thus, *Pleurotus ostreatus* has been found to degrade and mineralize xenobiotic compounds, such as PAHs, industrial dyes, and other soil pollutants, as described below (Cohen, Persky & Hadar, 2002). PAH substances have potent carcinogenic features and may be metabolized to bay-region diol epoxides, which are their ultimate carcinogenic forms. Unfortunately, currently encountered PAHs are common environmental pollutants that are strongly suspected of functioning as carcinogens to humans and the natural ecosystem (Rummel et al., 1999). Current studies include investigations of the biochemical mechanisms responsible for the degradation of these xenobiotics by different organisms and the enzymatic systems involved in these processes. White-rot fungi are a priority of most research interests for this purpose. Since the discovery that lignin is a natural polyaromatic compound that is degraded by ligninolytic extracellular oxidative enzymes from white-rot fungi, it is logical to hypothesise that these fungi can degrade PAHs using the same enzyme. Ligninolytic enzymes may reduce biotoxicity to the fungi and presumably also increase the availability of PAHs to facilitate degradation processes. During degradation, fungi can metabolize the initial PAH by cleaving the aromatic ring to form ring fission compounds with subsequent mineralization (Hammel et al., 1992; Nikiforova, Pozdnyakova & Turkovskaya, 2009). Prominent findings for the elimination of PAHs and other pollutants have shown in numerous studies targeting the genus *Pleurotus* for the biodegradation of PAHs. For example, Bezalel et al. (1996) have demonstrated the ability of *Pleurotus ostreatus* to degrade pollutants such as pyrene, anthracene, fluorene, and dibenzothiophene. According to their results, metabolites from pyrene, dibenzothiophene, anthracene, and fluorene amount from 45% up to 96% of the total organic-solvent-extractable metabolites, respectively. As a result, the authors concluded that the white-rot fungus *Pleurotus ostreatus* initially metabolises polycyclic aromatic hydrocarbons via the reactions. However, in contrast to non-ligninolytic fungi, *Pleurotus ostreatus* can mineralize these polycyclic aromatic hydrocarbons.

Adenipekun et al. (2015) studied the degradation of PAHs in spent and fresh cutting fluids (SCF and FCF) from contaminated soils and demonstrated that *Pleurotus ostreatus* degraded almost all of the PAH fraction to a greater extent than naphthalene in the fresh cutting fluid-contaminated soil. The degradation of PAHs in liquid cultures of some rot fungi, including *Pleurotus ostreatus*, has been studied by Schützendübel’s et al. (1999) group. During 7 weeks of mushroom cultivation, all PAHs uniformly removed, and fluorene, as well as anthracene, degraded more rapidly than other PAHs. Another chemical pollutant, chrysene, and its bioconversion by *Pleurotus ostreatus* D1 by cultivation conditions have been described by Nikiforova et al. (2010).

Recent studies of pollutant biodegradation have demonstrated a high potential capacity for the adsorption of PAH-contaminated water by cork waste (Jové et al., 2016). Following the PAH-adsorption process from water, the remaining cork waste should be treated and utilized. Thereby, according to the ability of some mushrooms to degrade PAHs, a new technology of contaminated cork utilization by some filamentous fungi, including *Pleurotus ostreatus*, was developed. In another report, Hadibarata & Teh (2014) *Pleurotus pulmonarius* strain F043 from tropical rainforest was used to degrade pyrene, a four-ring PAH in mineral medium broth. The maximum degradation level (90%) detected at pH 3, and the lowest level of PAH degradation (2%) observed at pH 10. Also, the degradation of pyrene increased from 2% to 96% when the temperature increased from 4 to 25 °C.

Descriptions of the biodegradation pathways of various PAHs have been provided by Cerniglia & Sutherland (2001), who examined the enzymatic mechanisms involved in the degradation of PAHs. Phase I and phase II enzymatic activities found in cell extracts of *Pleurotus ostreatus*. Cytochrome P-450 monooxygenase and epoxide hydrolase were found to oxidise and further hydrate phenanthrene. The involved enzymes are responsible for the initial attack of the aromatic ring in *Pleurotus ostreatus*, which leads to the mineralization of organic pollutants. An *ortho*-ring-cleavage activity of protocatechuate 3,4-dioxygenase was detected in the cytosolic fraction of *Pleurotus ostreatus* cell-free extracts and considered be responsible for the cleavage of the aromatic ring (Bezalel, Hadar & Cerniglia, 1997). *Pleurotus ostreatus* has an unusual combination of enzymes that catalyze the metabolism and degradation of PAHs through an initial oxidation mechanism similar to non-ligninolytic fungi and further activities of ring cleavage and mineralization similar to ligninolytic fungi. However, laccases of other white-rot fungi may also be involved in PAH oxidation via the mediation of laccase substrates (Johannes & Majcherczyk, 2000). In a study conducted using nutrient-rich liquid medium, the fungi exhibited potential in the decontamination of PAH-polluted soils (Eggen, 1999; Baldrian et al., 2000).

Another group of environmental pollutants is heavy metals from soil, water and different residues. Global ecosystem contamination with metals mostly is a result of progressing anthropogenic activity. Thereby, using mushrooms for mycoremediation of such kind pollutants gives a chance to decrease the anthropogenic pressure onto the environment through the natural sequestration process. Mycoremediation strategies provide suitable and eco-friendly solutions such as complete mineralization of the contaminants (Perelo, 2010). Among others, *Pleurotus* species can take out heavy metals from soil, such as Cu, Zn, Mn, and Fe, as it described on report (Boamponsem et al., 2013). In another work described the utilization of waste newspaper using a mycelium with *Pleurotus ostreatus* (Kopiński & Kwiatkowska-Marks, 2012). Newspaper waste and wheat straw mixture were composed for growing substrate. The maximum utilization was observed in the 3:1 mix of waste newspaper and wheat straw, according to the report. Presence of heavy metals in a substrate, such as Pb, also can effect on the mycelia growth and fruiting body production (Dulay et al., 2015). After examining five *Pleurotus* strains, decreasing of lowest mycelial growth was identified. Mercuri also has a negative influence on growth and fruit body production, as it has described for *Pleurotus tuber-regium* (Akpaja, Nwogu & Odibo, 2012). In general, mycoremediation baes on biosorption potential of different species. *Pleurotus* species tend to be a promising candidate as a biosorbent for heavy metals. According to the number of reports, a degree of tolerance varies among *Pleurotus* species for different heavy metals (Kapahi & Sachdeva, 2017).

## Conclusions

In this review, we have described research concerning the advantages of *Pleurotus* in industrial applications, ranging from reports about lignin decomposition to straw waste and phenolic compound degradation, dye-decolourising ability, nutritional content values, and other investigations. All the studies suggest the significant potential of the utility and profitability of *Pleurotus* spp. However, despite extensive research on its biotechnological applications (in agro-business, pollutant biodegradation, and life sciences), a large portion of the biochemical pathways that make the genus *Pleurotus* an attractive biological target for future research purposes remain undescribed.

Additionally, various highly effective methods for studying fungal genomic variability are applied using various systems of genetic transformation. The modification of genes through their transformation to create fungal mutant lines is an essential step in investigations of gene function and relationships (Poyedinok & Blume, 2018). Such strategies will facilitate analyses of fungal genetic classification and improve the biosynthetic activity of many biotechnologically important *Pleurotus* strains.

Here we address the economic feasibility of the technologies described above in large scale; only mushroom production could be considered as a successful implementation of agricultural residues biodegradation. In order to realize the full potential and use the circular economy approach, we need to mention the low technology readiness level of the potential of these technologies for biorefinery such as, for example, enzymatic decomposition of lignocellulose to valuable chemicals. Moreover, there are limited market opportunities that do not allow to estimate investment and manufacturing cost. The estimation of the potential of the spent mushroom substrate for biorefinery is only under technological development. In our other study related to shiitake cultivation, we developed an approach where the total mass balance was applied, and the potential for biorefinery was estimated (Xiong et al., 2019).

## Additional Information and Declarations

### Data Availability

The following information was supplied regarding data availability: The research in this article did not generate any data or code. The sequence of analysis is included in the article.

## References

[ref-1] Adenipekun CO, Ipeaiyeda AR, Olayonwa AJ, Egbewale SO (2015). Biodegradation of polycyclic aromatic hydrocarbons (PAHs) in spent and fresh cutting fluids contaminated soils by *Pleurotus pulmonarius* (Fries). Quelet and *Pleurotus ostreatus* (Jacq.) Fr. P. Kumm. African Journal of Biotechnology.

[ref-2] Ahmed SA, Kadam JA, Mane VP, Patil SS, Baig MMV (2008). Biological efficiency and nutritional contents of *Pleurotus florida* (Mont.) singer cultivated on different agro-wastes. Nature and Science of Sleep.

[ref-3] Akpaja EO, Nwogu NA, Odibo EA (2012). Effect of some heavy metals on the growth and development of *Pleurotus tuber-regium*. Mycosphere.

[ref-4] Alves RP, Bolson SM, De Albuquerque MP, De Carvalho Victori F, Pereira AB (2017). A Potential use of edible mushrooms *Pleurotus ostreatoroseus* singer (*Pleurotaceae*) and *Lentinus sajor-caju* (Fr.) Fr. (Polyporaceae) in metal remediation processes. Revista de Biologia Neotropical.

[ref-5] Autore F, Del Vecchio C, Fraternali F, Giardina P, Sannia G, Faraco V (2009). Molecular determinants of peculiar properties of a *Pleurotus ostreatus* laccase: analysis by site-directed mutagenesis. Enzyme and Microbial Technology.

[ref-6] Balan V, Da Costa Sousa L, Chundawat SPS, Vismeh R, Jones AD, Dale BE (2008). Mushroom spent straw: a potential substrate for an ethanol-based biorefinery. Journal of Industrial Microbiology & Biotechnology.

[ref-7] Baldrian P, Gabriel J (2002). Copper and cadmium increase laccase activity in *Pleurotus ostreatus*. FEMS Microbiology Letters.

[ref-8] Baldrian P, in Der Wiesche C, Gabriel J, Nerud F, Zadrazil F (2000). Influence of cadmium and mercury on activities of ligninolytic enzymes and degradation of polycyclic aromatic hydrocarbons by *Pleurotus ostreatus* in soil. Applied and Environmental Microbiology.

[ref-9] Baldrian P, Valásková V, Merhautová V, Gabriel J (2005). Degradation of lignocellulose by *Pleurotus ostreatus* in the presence of copper, manganese, lead and zinc. Research in Microbiology.

[ref-10] Bánfia R, Pohnera Z, Kovácsb J, Luzicsa S, Nagyc A, Dudásc M, Tanosb P, Márialigetia K, Vajnaa B (2015). Characterisation of the large-scale production process of oyster mushroom (*Pleurotus ostreatus*) with the analysis of succession and spatial heterogeneity of lignocellulolytic enzyme activities. Fungal Biology.

[ref-11] Bao D, Aimi T, Kitamoto Y (2005). Cladistic relationships among the *Pleurotus ostreatus* complex, the *Pleurotus pulmonarius* complex, and *Pleurotus eryngii* based on the mitochondrial small subunit ribosomal DNA sequence analysis. Journal of Wood Science.

[ref-12] Bazanella GC, Vaz Araujo C, Castoldi R, Maciel GM, Inácio F, Marques de Souza C, Bracht A, Peralta R, Bazanella G (2013). Ligninolytic enzymes from white-rot fungi and application in the removal of synthetic dyes. Fungal Enzymes.

[ref-13] Bellettini MB, Fiorda FA, Maieves HA, Teixeira GL, Ávila S, Hornung PS, Júnior AM, Ribani RH (in press). Factors affecting mushroom *Pleurotus* spp. Saudi Journal of Biological Sciences.

[ref-14] Bettin F, Montanari Q, Calloni R, Gaio TA, Silveira MM, Dillon AJP (2009). Production of laccases in submerged process by *Pleurotus sajor-caju* PS-2001 in relation to carbon and organic nitrogen sources, antifoams and Tween 80. Journal of Industrial Microbiology & Biotechnology.

[ref-15] Bezalel L, Hadar Y, Cerniglia CE (1997). Enzymatic mechanisms involved in phenanthrene degradation by the white rot fungus *Pleurotus ostreatus*. Applied and Environmental Microbiology.

[ref-16] Bezalel L, Hadar Y, Fu PP, Freeman JP, Cerniglia CE (1996). Initial oxidation products in the metabolism of pyrene, anthracene, fluorene, and dibenzothiophene by the white rot fungus *Pleurotus ostreatus*. Applied and Environmental Microbiology.

[ref-152] Boamponsem GA, Obeng AK, Osei-Kwateng M, Badu AO (2013). Accumulation of heavy metals by *pleurotus ostreatus* from soils of metal scrap sites. IJCRR.

[ref-17] Böckle B, Martínez MJ, Guillén F, Martínez ÁT (1999). Mechanism of peroxidase inactivation in liquid cultures of the ligninolytic fungus *Pleurotus pulmonarius*. Applied and Environmental Microbiology.

[ref-18] Bourbonnais R, Paice MG, Reid ID, Lanthier P, Yaguchi M (1995). Lignin oxidation by laccase isozymes from *Trametes versicolor* and role of the mediator 2,2′-azinobis(3-ethylbenzthiazoline-6-sulfonate) in kraft lignin depolymerization. Applied and Environmental Microbiology.

[ref-19] Camarero S, Bockle B, Martinez MJ, Martinez AT (1996). Manganese-mediated lignin degradation by *Pleurotus pulmonarius*. Applied and Environmental Microbiology.

[ref-20] Camarero S, Ruiz-Dueñas FJ, Sarkar S, Martínez MJ, Martínez AT (2000). The cloning of a new peroxidase found in lignocellulose cultures of *Pleurotus eryngii* and sequence comparison with other fungal peroxidases. FEMS Microbiology Letters.

[ref-21] Camarero S, Sarkar S, Ruiz-Dueñas FJ, Martínez MJ, Martínez AT (1999). Description of a versatile peroxidase involved in the natural degradation of lignin that has both manganese peroxidase and lignin peroxidase substrate interaction sites. Journal of Biological Chemistry.

[ref-22] Camassola M, Dillon A (2009). Biological pretreatment of sugar cane bagasse for the production of cellulases and xylanases by *Penicillium echinulatum*. Industrial Crops and Products.

[ref-23] Centre for Monitoring Indian Economy (CMIE) (2001). Centre for Monitoring Indian Economy. https://www.cmie.com/.

[ref-24] Cerniglia CE, Sutherland JB, Gadd GM (2001). Bioremediation of polycyclic aromatic hydrocarbons by ligninolytic and non-ligninolytic fungi. Fungi in Bioremediation.

[ref-25] Choi DB, Ding JL, Cha W-S (2007). Homology search of genus *Pleurotus* using an internal transcribed spacer region. Korean Journal of Chemical Engineering.

[ref-102] Christopher LP, Yao B, Ji Y (2014). Lignin biodegradation with laccase-mediator systems. Frontiers in Energy Research.

[ref-26] Cohen R, Persky L, Hadar Y (2002). Biotechnological applications and potential of wood-615degrading mushrooms of the genus *Pleurotus*. Applied Microbiology and Biotechnology.

[ref-27] Da Silva MCS, Naozuka J, Da Luz JMR, De Assunção LS, Oliveira PV, Vanetti MCD, Bazzolli DMS, Kasuya MCM (2012). Enrichment of *Pleurotus ostreatus* mushrooms with selenium in coffee husks. Food Chemistry.

[ref-28] Deepalakshmi K, Mirunalini S (2014). *Pleurotus ostreatus*: an oyster mushroom with nutritional and medicinal properties. Journal of Biochemical Technology.

[ref-29] Dulay RMR, De Castro MAEG, Coloma NB, Bernardo AP, Cruz AGD, Tiniola RC, Kalaw SP, Reyes RG (2015). Effects and myco-remediation of lead (Pb) in five *Pleurotus* mushrooms. International Journal of Biology, Pharmacy and Allied Sciences.

[ref-30] Eggen T (1999). Application of fungal substrate from commercial mushroom production—*Pleuorotus ostreatus*—for bioremediation of creosote contaminated soil. International Biodeterioration & Biodegradation.

[ref-31] Elisashvili V, Penninckx M, Kachlishvili E, Asatiani M, Kvesitadze G (2006). Use of *Pleurotus dryinus* for lignocellulolytic enzymes production in submerged fermentation of mandarin peels and tree leaves. Enzyme and Microbial Technology.

[ref-32] Elisashvili V, Penninckx M, Kachlishvili E, Tsiklauri N, Metreveli E, Kharziani T, Kvesitadze G (2008). *Lentinus edodes* and *Pleurotus* species lignocellulolytic enzymes activity in submerged and solid-state fermentation of lignocellulosic wastes of different composition. Bioresource Technology.

[ref-33] Eriksson K-EL, Blanchette RA, Ander P (1990). Microbial and enzymatic degradation of wood and wood components.

[ref-34] Estrada AER, Jimenez-Gasco MDM, Royse DJ (2010). *Pleurotus eryngii* species complex: sequence analysis and phylogeny based on partial EF1α and RPB2 genes. Fungal Biology.

[ref-35] Fernández-Fueyo E, Castanera R, Ruiz-Dueñas FJ, López-Lucendo MF, Ramírez L, Pisabarro AG, Martínez AT (2014). Ligninolytic peroxidase gene expression by Pleurotus ostreatus: Differential regulation in lignocellulose medium and effect of temperature and pH. Fungal Genetics and Biology.

[ref-36] Galbe M, Zacchi G (2007). Pretreatment of lignocellulosic materials for efficient bioethanol production. Advances in Biochemical Engineering/Biotechnology.

[ref-37] Garber RC, Yoder OC (1983). Isolation of DNA from filamentous fungi and separation into nuclear, mitochondrial, ribosomal, and plasmid components. Analytical Biochemistry.

[ref-38] Garcia-Ruiz E, Gonzalez-Perez D, Ruiz-Dueñas FJ, Martínez AT, Alcalde M (2012). Directed evolution of a temperature-, peroxide- and alkaline pH-tolerant versatile peroxidase. Biochemical Journal.

[ref-39] Giardina P, Palmieri G, Fontanella B, Rivieccio V, Sannia G (2000). Manganese peroxidase isoenzymes produced by *Pleurotus ostreatus* grown on wood sawdust. Archives of Biochemistry and Biophysics.

[ref-40] Golak-Siwulska I, Kałużewicz A, Spiżewski T, Siwulski M, Sobieralski K (2018). Bioactive compounds and medicinal properties of Oyster mushrooms (*Pleurotus* sp.). Folia Horticulturae.

[ref-42] Gonzalez P, Labarère J (2000). Phylogenetic relationships of *Pleurotus* species according to the sequence and secondary structure of the mitochondrial small-subunit rRNA V4, V6 and V9 domains. Microbiology.

[ref-43] Gregori A, Svagelj M, Pohleven J (2007). Cultivation techniques and medicinal properties of *Pleurotus* spp. Food Technology and Biotechnology.

[ref-44] Gucia M, Jarzyńska G, Rafał E, Roszak M, Kojta AK, Osiej I, Falandysz J (2012). Multivariate analysis of mineral constituents of edible Parasol Mushroom (*Macrolepiota procera*) and soils beneath fruiting bodies collected from Northern Poland. Environmental Science and Pollution Research.

[ref-45] Guerriero G, Hausman J-F, Strauss J, Ertan H, Siddiqui KS (2016). Lignocellulosic biomass: biosynthesis, degradation, and industrial utilization. Engineering in Life Sciences.

[ref-46] Hadar Y, Kerem Z, Gorodecki B, Ardon O (1992). Utilization of lignocellulosic waste by the edible mushroom, *Pleurotus*. Biodegradation.

[ref-47] Hadibarata T, Teh ZC (2014). Optimization of pyrene degradation by white-rot fungus *Pleurotus pulmonarius* F043 and characterization of its metabolites. Bioprocess and Biosystems Engineering.

[ref-48] Hammel KE, Gai WZ, Green B, Moen MA (1992). Oxidative degradation of phenanthrene by the ligninolytic fungus *Phanerochaete chrysosporium*. Applied and Environmental Microbiology.

[ref-49] Hatakka A (1983). Pretreatment of wheat straw by white-rot fungi for enzymic saccharification of cellulose. European Journal of Applied Microbiology and Biotechnology.

[ref-50] Hatakka A, Hammel KE, Hofrichter M (2011). Fungal Biodegradation of Lignocelluloses. Industrial Applications.

[ref-51] Hawksworth D, Kirk P, Sutton BC, Pegler DN (1996). Ainsworth & Bisby’s dictionary of the Fungi. Revista Do Instituto De Medicina Tropical De Sao Paulo.

[ref-52] Higuchi T (2004). Microbial degradation of lignin: role of lignin peroxidase, manganese peroxidase, and laccase. Proceedings of the Japan Academy Series B-physical and Biological Sciences.

[ref-53] Hilber O (1982). Die Gattung Pleurotus (Fr.) Kummer: unter besonderer Berücksichtigung des Pleurotus eryngii-Formenkomplexes.

[ref-54] Hildén K, Martinez AT, Hatakka A, Lundell T (2005). The two manganese peroxidases Pr-MnP2 and Pr-MnP3 of *Phlebia radiata*, a lignin-degrading basidiomycete, are phylogenetically and structurally divergent. Fungal Genetics and Biology.

[ref-55] Hirano T, Honda Y, Watanabe T, Kuwahara M (2000). Degradation of bisphenol A by the lignin-degrading enzyme, manganese peroxidase, produced by the white-rot basidiomycete, *Pleurotus ostreatus*. Bioscience, Biotechnology, and Biochemistry.

[ref-56] Hofrichter M (2002). Review: lignin conversion by manganese peroxidase (MnP). Enzyme and Microbial Technology.

[ref-57] Honda Y, Matsuyama T, Irie T, Watanabe T, Kuwahara M (2000). Carboxin resistance transformation of the homobasidiomycete fungus *Pleurotus ostreatus*. Current Genetics.

[ref-58] Hoopes JT, Dean JF (2004). Ferroxidase activity in a laccase-like multicopper oxidase from *Liriodendron tulipifera*. Plant physiology and Biochemistry.

[ref-59] Huttermann A, Hamza A, Chet I, Majcherczyk A, Fouad T, Badr A, Cohen R, Persky L, Hadar Y (2000). Recycling of agricultural wastes by white-rot fungi for the production of fodder for ruminants. Agro Food Industry Hi-Tech.

[ref-60] Irie T, Honda Y, Watanabe T, Kuwahara M (2001). Homologous expression of recombinant manganese peroxidase genes in ligninolytic fungus *Pleurotus ostreatus*. Applied Microbiology and Biotechnology.

[ref-61] Isroi I, Millati R, Syamsiah S, Niklasson C, Cahyanto MN, Ludquist K, Taherzadeh MJ (2011). Biological pretreatment of lignocelluloses with white-rot fungi and its applications: a review. BioResources.

[ref-62] Johannes C, Majcherczyk A (2000). Natural mediators in the oxidation of polycyclic aromatic hydrocarbons by laccase mediator systems. Applied and Environmental Microbiology.

[ref-63] Jové P, Olivella MÀ, Camarero S, Caixach J, Planas C, Cano L, De Las Heras FX (2016). Fungal biodegradation of anthracene-polluted cork: a comparative study. Journal of Environmental Science and Health. Part A, Toxic/Hazardous Substances & Environmental Engineering.

[ref-64] Kalač P (2016). Edible mushrooms: chemical composition and nutritional value.

[ref-65] Kapahi M, Sachdeva S (2017). Mycoremediation potential of *Pleurotus* species for heavy metals: a review. Bioresources and Bioprocessing.

[ref-151] Kempken F (2013). Agricultural applications.

[ref-66] Kerem Z, Friesem D, Hadar Y (1992). Lignocellulose degradation during solid-state fermentation: *Pleurotus ostreatus* versus *Phanerochaete chrysosporium*. Applied and Environmental Microbiology.

[ref-67] Kim S, Dale BE (2004). Global potential bioethanol production from wasted crops and crop residues. Biomass and Bioenergy.

[ref-68] Kirk TK, Farrell RL (1987). Enzymatic “combustion”: the microbial degradation of lignin. Annual Review of Microbiology.

[ref-69] Knop D, Levinson D, Makovitzki A, Agami A, Lerer E, Mimran A, Yarden O, Hadar Y (2016). Limits of versatility of versatile peroxidase. Applied and Environmental Microbiology.

[ref-70] Kopiński L, Kwiatkowska-Marks S (2012). Utilization of waste newspaper using oyster mushroom mycelium. Industrial & Engineering Chemistry Research.

[ref-71] Lauradó G, Morris HJ, Tamayo V, Lebeque Y, Beltran Y, Marcos J, Moukha S, Creppy EE, Bermudez RC (2015). Haematopoiesis radioprotection in Balb/c mice by an aqueous mycelium extract from the Basidiomycete *Pleurotus ostreatus* mushroom. Natural Products Research.

[ref-72] Leonowicz A, Cho NS, Luterek J, Wilkolazka A, Wojtas-Wasilewska M, Matuszewska A, Hofrichter M, Wesenberg D, Rogalski J (2001). Fungal laccase: properties and activity on lignin. Journal of Basic Microbiology.

[ref-73] Li J, He X, Liu H-B, Yang ZhL, Zhao Zh-W (2017). Species clarification of oyster mushrooms in China and their DNA barcoding. Mycological Progress.

[ref-75] Ma KH, Lee GA, Lee SY, Gwag JG, Kim TS, Kong WS, Seo KI, Lee GS, Park YJ (2009). Development and characterization of new microsatellite markers for the oyster mushroom (*Pleurotus ostreatus*). Journal of Microbiology and Biotechnology.

[ref-76] Marchetti R, Vasmara C, Florio G (2016). Biomethanation potential of wetland biomass in codigestion with pig slurry. Waste and Biomass Valorization.

[ref-77] Martínez MJ, Ruiz-Dueñas FJ, Guillén F, Martínez AT (1996). Purification and catalytic properties of two manganese peroxidase isoenzymes from *Pleurotus eryngii*. European Journal of Biochemistry.

[ref-78] Martínez AT, Ruiz-Dueñas FJ, Martínez MJ, Del Río JC, Gutiérrez A (2009). Enzymatic delignification of plant cell wall: from nature to mill. Current Opinion in Biotechnology.

[ref-79] Mau J-L, Lin Y-P, Chen P-T, Wu Y-H, Peng J-T (1998). Flavor compounds in King Oyster Mushrooms *Pleurotus eryngii*. Journal of Agricultural and Food Chemistry.

[ref-80] Mester T, Field JA (1998). Characterization of a novel manganese peroxidase-lignin peroxidase hybrid isozyme produced by *Bjerkandera* species strain BOS55 in the absence of manganese. Journal of Biological Chemistry.

[ref-81] Mikiashvili N, Wasser SP, Nevo E, Elisashvili V (2006). Effects of carbon and nitrogen sources on *Pleurotus ostreatus* ligninolytic enzyme activity. World Journal of Microbiology and Biotechnology.

[ref-82] Mohamed EM, Farghaly FA (2014). Bioactive compounds of fresh and dried *Pleurotus ostreatus* Mushroom. International Journal of Biotechnology for Wellness Industries.

[ref-83] Morales M, Mate MJ, Romero A, Martínez MJ, Martínez ÁT, Ruiz-Dueñas FJ (2012). Two oxidation sites for low redox potential substrates: a directed mutagenesis, kinetic, and crystallographic study on *Pleurotus eryngii* versatile peroxidase. Journal of Biological Chemistry.

[ref-84] Moreira PR, Duez C, Dehareng D, Antunes A, Almeida-Vala E, Frere JM, Malcata FX, Duarte JC (2005). Molecular characterization of a versatile peroxidase from a *Bjerkandera strain*. Journal of Biotechnology.

[ref-85] Morris HJ, Beltran Y, Llaurado G, Batista PL, Perraud-Gaime I, Garcia N, Moukha S, Bermúdez RC, Cos P, Hernández E, Diez JC (2017). Mycelia from *Pleurotus* sp. (oyster mushroom): a new wave of antimicrobials, anticancer and antioxidant bioingredients. International Journal of Phytocosmetics and Natural Ingredients.

[ref-86] Moussa TAA (2009). Molecular characterization of the phenol oxidase (*pox2*) gene from the ligninolytic fungus *Pleurotus ostreatus*. FEMS Microbiology Letters.

[ref-87] Müller HW, Trösch W (1986). Screening of white-rot fungi for biological pretreatment of wheat straw for biogas production. Applied Microbiology and Biotechnology.

[ref-88] Munari F, Aparecida Gaio T, Dillon A (2009). Phenol degradation and colour removal in submerged culture of *Pleurotus sajor-caju* with paper mill eluents. Biocatalysis and Biotransformation.

[ref-89] Munari FM, Gaio TA, Calloni R, Dillon AJP (2008). Decolorization of textile dyes by enzymatic extract and submerged cultures of *Pleurotus sajor-caju*. World Journal of Microbiology and Biotechnology.

[ref-90] Mustafa A, Poulsen T, Sheng K (2016). Fungal pretreatment of rice straw with *Pleurotus ostreatus* and *Trichoderma reesei* to enhance methane production under solid-state anaerobic digestion. Applied Energy.

[ref-91] Myronycheva O, Bandura I, Bisko N, Gryganskyi AP, Karlsson O (2017). Assessment of the growth and fruiting of 19 oyster mushroom strains for indoor cultivation on lignocellulosic wastes. BioResources.

[ref-92] Nakazawa T, Izuno A, Kodera R, Miyazaki Y, Sakamoto M, Isagi Y, Honda Y (2017). Identification of two mutations that cause defects in the ligninolytic system through an efficient forward genetics in the white‐rot agaricomycete *Pleurotus ostreatus*. Environmental Microbiology.

[ref-93] Nguyen HDT, Seifert KA (2008). Description and DNA barcoding of three new species of *Leohumicola* from South Africa and the United States. Persoonia.

[ref-94] Nikiforova SV, Pozdnyakova NN, Makarov OE, Chernyshova MP, Turkovskaya OV (2010). Chrysene bioconversion by the white rot fungus *Pleurotus ostreatus* D1. Microbiology.

[ref-95] Nikiforova SV, Pozdnyakova NN, Turkovskaya OV (2009). Emulsifying agent production during PAHs degradation by the white rot fungus *Pleurotus ostreatus* D1. Current Microbiology.

[ref-96] Oloke JK, Adebayo EA (2015). Effectiveness of immunotherapies from oyster mushroom (*Pleurotus* species) in the management of immunocompromised patients. International Journal of Immunological Studies.

[ref-97] Palma C, Lloret L, Sepúlveda L, Contreras E (2016). Production of versatile peroxidase from *Pleurotus eryngii* by solid-state fermentation using agricultural residues and evaluation of its catalytic properties. Preparative Biochemistry & Biotechnology.

[ref-98] Palmieri G, Giardina P, Bianco C, Fontanella B, Sannia G (2000). Copper induction of laccase isoenzymes in the ligninolytic fungus *Pleurotus ostreatus*. Applied and Environmental Microbiology.

[ref-99] Patel H, Gupte S, Gahlout M, Gupte A (2014). Purification and characterization of an extracellular laccase from solid-state culture of *Pleurotus ostreatus* HP-1. 3 Biotechnology.

[ref-100] Patil SS, Ahmed SA, Telang SM, Baig MM (2010). The nutritional value of *Pleurotus ostreatus* Kumm. Cultivated on different lignocellulosic agro-wastes. Innovative Romanian Food Biotechnology.

[ref-101] Pathak R, Joshi N, Dwivedi RR (2009). Eco-friendly production of *Agaricus bisporus* (lange) imbach (white button mushroom). Natural Science.

[ref-103] Pawlik A, Janusz G, Koszerny J, Małek W, Rogalski J (2012). Genetic diversity of the edible mushroom *Pleurotus* sp. by amplified fragment length polymorphism. Current Microbiology.

[ref-104] Perelo LW (2010). Review: in situ and bioremediation of organic pollutants in aquatic sedments. Journal of Hazardous Materials.

[ref-105] Piska K, Ziaja K, Muszynska B (2016). Edible mushroom *Pleurotus ostreatus* (Oyster mushroom)—its dietary significance and biological activity. Acta scientiarum Polonorum. Hortorum cultus=Ogrodnictwo.

[ref-106] Poyedinok NL, Blume YB (2018). Advances, problems, and prospects of genetic transformation of fungi. Cytology and Genetics.

[ref-107] Pozdnyakova NN, Jarosz-Wilkolazka A, Polak J, Grąz M, Turkovskaya OV (2015). Decolourisation of anthraquinone-and anthracene-type dyes by versatile peroxidases from *Bjerkandera fumosa* and *Pleurotus ostreatus* D1. Biocatalysis and Biotransformation.

[ref-108] Pozdnyakova N, Rodakiewicz-Nowak J, Turkovskaya O, Haber J (2006). Oxidative degradation of polyaromatic hydrocarbons catalyzed by blue laccase from *Pleurotus ostreatus* D1 in the presence of synthetic mediators. Enzyme and Microbial Technology.

[ref-109] Reddy G, Ravindra Babu P, Komaraiah P, Roy KRRM, Kothari IL (2003). Utilization of banana waste for the production of lignolytic and cellulolytic enzymes by solid substrate fermentation using two *Pleurotus* species (*P. ostreatus* and *P. sajo-caju*). Process Biochemistry.

[ref-110] Rummel AM, Trosko JE, Wilson MR, Upham BL (1999). Polycyclic aromatic hydrocarbons with bay-like regions inhibited gap junctional intercellular communication and stimulated MAPK activity. Toxicological Sciences.

[ref-111] Sáez-Jiménez V, Acebes S, Guallar V, Martínez AT, Ruiz-Dueñas FJ (2015). Improving the oxidative stability of a high redox potential fungal peroxidase by rational design. PLOS ONE.

[ref-112] Salame TM, Knop D, Levinson D, Mabjeesh SJ, Yarden O, Hadar Y (2014). Inactivation of a *Pleurotus ostreatus* versatile peroxidase-encoding gene (mnp2) results in reduced lignin degradation. Environmental Microbiology.

[ref-113] Sánchez C (2010). Cultivation of *Pleurotus ostreatus* and other edible mushrooms. Applied Microbiology and Biotechnology.

[ref-114] Sarkar S, Martínez AT, Martínez MJ (1997). Biochemical and molecular characterization of a manganese peroxidase isoenzyme from *Pleurotus ostreatus*. Biochimica Et Biophysica Acta.

[ref-115] Schüttmann I, Bouws H, Szweda R, Suckow M, Czermak P, Zorn H (2014). Induction, characterization, and heterologous expression of a carotenoid degrading versatile peroxidase from *Pleurotus sapidus*. Journal of Molecular Catalysis B: Enzymatic.

[ref-116] Schützendübel A, Majcherczyk A, Johannes C, Hüttermann A (1999). Degradation of fluorene, anthracene, phenanthrene, fluoranthene, and pyrene lacks connection to the production of extracellular enzymes by *Pleurotus ostreatus* and *Bjerkandera adusta*. International Biodeterioration and Biodegradation.

[ref-117] Seif E, Leigh J, Liu Y, Roewer I, Forget L, Lang BF (2005). Comparative mitochondrial genomics in zygomycetes: bacteria-like RNase P RNAs, mobile elements and a close source of the group I intron invasion in angiosperms. Nucleic Acids Research.

[ref-118] Seifert KA, Samson RA, Dewaard JR, Houbraken J, Lévesque CA, Moncalvo J-M, Louis-Seize G, Hebert PDN (2007). Prospects for fungus identification using CO1 DNA barcodes, with *Penicillium* as a test case. Proceedings of the National Academy of Sciences of the United States of America.

[ref-119] Selegean M, Putz MV, Rugea T (2009). Effect of the polysaccharide extract from the edible mushroom *Pleurotus ostreatus* against infectious bursal disease virus. International Journal of Molecular Sciences.

[ref-120] Shnyreva A, Kozhevnikova EYV, Barkov A, Shnyreva A (2017). Solid-state cultivation of edible oyster mushrooms, *Pleurotus* spp. under laboratory conditions. Advances in Microbiology.

[ref-121] Shnyreva AA, Shnyreva AV (2015). Phylogenetic analysis of *Pleurotus* species. Genetika.

[ref-122] Silanikove N, Danai O, Levanon D (1988). Composted cotton straw silage as a substrate for *Pleurotus* sp. cultivation. Biological Wastes.

[ref-123] Skočaj M, Gregori A, Grundner M, Sepčić K, Sežun M (2018). Hydrolytic and oxidative enzyme production through cultivation of *Pleurotus ostreatus* on pulp and paper industry wastes. Holzforschung.

[ref-124] Snajdr J, Baldrian P (2007). Temperature affects the production, activity and stability of ligninolytic enzymes in *Pleurotus ostreatus* and *Trametes versicolor*. Folia Microbiologica.

[ref-125] Soden DM, Dobson AD (2001). Differential regulation of laccase gene expression in *Pleurotus sajor-caju*. Microbiology.

[ref-126] Stajić M, Persky L, Friesem D, Hadar Y, Wasser SP, Nevo E, Vukojević J (2006). Effect of different carbon and nitrogen sources on laccase and peroxidases production by selected *Pleurotus* species. Enzyme and Microbial Technology.

[ref-127] Stamets P (2011). Growing gourmet and medicinal mushrooms.

[ref-128] Thurston CF (1994). The structure and function of fungal laccases. Microbiology.

[ref-129] Tian X, Fang Z, Guo F (2012). Impact and prospective of fungal pre-treatment of lignocellulosic biomass for enzymatic hydrolysis. Biofuels, Bioproducts and Biorefining.

[ref-130] Tinoco R, Acevedo A, Galindo E, Serrano-Carreón L (2011). Increasing *Pleurotus ostreatus* laccase production by culture medium optimization and copper/lignin synergistic induction. Journal of Industrial Microbiology and Biotechnology.

[ref-131] Tsukihara T, Honda Y, Watanabe T, Watanabe T (2006). Molecular breeding of white rot fungus *Pleurotus ostreatus* by homologous expression of its versatile peroxidase MnP2. Applied Microbiology and Biotechnology.

[ref-132] Tsuneda A, Thorn RG (2011). Interactions of wood decay fungi with other microorganisms, with emphasis on the degradation of cell walls. Canadian Journal of Botany.

[ref-133] Urbanelli S, Rosa VD, Punelli F, Porretta D, Reverberi M, Fabbri AA, Fanelli C (2007). DNA-fingerprinting (AFLP and RFLP) for genotypic identification in species of the *Pleurotus eryngii* complex. Applied Microbiology and Biotechnology.

[ref-134] Van Aken B, Hofrichter M, Scheibner K, Hatakka AI, Naveau H, Agathos SN (1999). Transformation and mineralization of 2,4,6-trinitrotoluene (TNT) by manganese peroxidase from the white-rot basidiomycete *Phlebia radiata*. Biodegradation.

[ref-135] Vandendries R (1933). De la valeur du barrage sexuel, comme critérium dans l’analyse d’une sporée tétrapolaire de basidiomycète: *Pleurotus ostreatus*. Genetica.

[ref-136] Vasmara C, Cianchetta S, Marchetti R, Galletti S (2015). Biogas production from wheat straw pre-treated with ligninolytic fungi and co-digestion with pig slurry. Environmental Engineering and Management Journal.

[ref-137] Venturella G, Gargano M, Compagno R (2015). The genus *Pleurotus* in Italy. Flora Mediterranea.

[ref-138] Vilgalys R, Moncalvo JM, Liou SR, Volovsek M, Royse DJ (1996). Recent advances in molecular systematics of the genus “*Pleurotus”*. World Society for Mushroom Biology and Mushroom Products.

[ref-139] Vilgalys R, Sun BL (1994). Ancient and recent patterns of geographic speciation in the oyster mushroom *Pleurotus* revealed by phylogenetic analysis of ribosomal DNA sequences. Proceedings of the National Academy of Sciences of the United States of America.

[ref-140] Wang Y, Zeng F, Hon CC, Zhang Y, Leung FCC (2008). The mitochondrial genome of the Basidiomycete fungus *Pleurotus ostreatus* (oyster mushroom). FEMS Microbiology Letters.

[ref-141] Wesenberg D, Kyriakides I, Agathos SN (2003). White-rot fungi and their enzymes for the treatment of industrial dye effluents. Biotechnology Advances.

[ref-142] Widsten P, Kandelbauer A (2008). Laccase applications in the forest products industry: a review. Enzyme and Microbial Technology.

[ref-143] Wong DWS (2009). Structure and action mechanism of ligninolytic enzymes. Applied Biochemistry and Biotechnology.

[ref-144] Wu J, Xiao YZ, Yu HQ (2005). Degradation of lignin in pulp mill wastewaters by white-rot fungi on biofilm. Bioresource Technology.

[ref-145] Xiong S, Martín C, Eilertsen L, Wei M, Myronycheva O, Larsson SH, Lestander TA, Atterhem L, Jönsson LJ (2019). Energy-efficient substrate pasteurisation for combined production of shiitake mushroom (*Lentinula edodes*) and bioethanol. Bioresource Technology.

[ref-146] Yan P-S, Jiang J-H (2005). Preliminary research of the RAPD molecular marker-assisted breeding of the edible basidiomycete *Stropharia rugoso-annulata*. World Journal of Microbiology and Biotechnology.

[ref-147] Zervakis G, Moncalvo J-M, Vilgalys R (2004). Molecular phylogeny, biogeography and speciation of the mushroom species *Pleurotus cystidiosus* and allied taxa. Microbiology.

[ref-148] Zervakis G, Sourdis J, Balis C (1994). Genetic variability and systematics of eleven *Pleurotus* species based on isozyme analysis. Mycological Research.

[ref-149] Zhong W, Zhang Z, Luo Y, Sun S, Qiao W, Xiao M (2011). Effect of biological pretreatments in enhancing corn straw biogas production. Bioresource Technology.

[ref-150] Zou YJ, Wang HX, Zhang JH (2018). A novel peroxidase from fresh fruiting bodies of the mushroom *Pleurotus pulmonarius*. International Journal of Peptide Research and Therapeutics.

